# Monotony in Farm Life

**Published:** 1891-05

**Authors:** 


### MONOTONY IN FARM LIFE.

That "variety is the spice of life" is very generally admitted, says the *Country Gentleman.* Yet the farmers, as a class, do not get as much of the spice of variety as they should. Their lives are often monotonous almost beyond endurance. It must be confessed, however, that farmers are themselves largely to blame for this monotony. The isolation of the farmer's house is one of the greatest obstacles in the way of social enjoyment he has to deal with. It is our duty to one another to cultivate social habits, to visit each other's homes, compare notes, and make suggestions as to the best methods of farming, etc. Nor does our duty end at this neighborly intercourse. Our first obligations, like charity, begin at home. We should make the farm home just as attractive as our means will allow.

How many farm homes are utterly devoid of attraction! The house is unpainted and uninviting; the fences are dilapidated; the gates are sagged down to the ground; the entire surroundings are dreary in the extreme. In the yard grow noxious weeds where roses and other ornamental plants should be seen. Scrubby bushes stand where a neat grass plot should be. Instead of an orchard of beautiful fruit trees, we see here and there at long intervals, a few broken-limbed, neglected apple and peach trees, and a dense thicket of bushes hard by.

The adjacent fields have been cultivated for years in the same crops plowed by the same shallow running plows and producing their steadily decreasing little crops of corn and cotton. No manure has ever been applied since the land was first cleared. The same round of drudgery has been gone through with year after year; the same cry of "hard times" has always been heard on this farm, and the same doleful expression has been seen on the farmer's face for years past. Indeed, this lugubrious expression seems to have settled down to stay, and stay it doubtless will until the grave shall close over his weary form.

The work stock receives but a scanty and monotonous ration of corn and fodder, while the milk cows have to subsist on what they can glean from the bare forests and old fields.

Is it at all strange that boys should abandon this kind of sordid, wearisome life and flee to the city as soon as they become of age? It would indeed be strange if they remained there.

But think of the hardships the poor wife must endure upon such a cheerless farm as this. Oftentimes without even the common luxury of a vegetable garden, and, perchance, little or no poultry or milk to aid her in her culinary cares. Is it not enough to run a sensitive woman crazy? There are thousands of just such dreary, monotonous, cheerless farm houses in the west and south, and perhaps elsewhere. Executive ability is not confined to men, by any means. I know plenty of women who have more practical common sense in a minute than their incapable husbands will have in a lifetime. Some women should hold the tiller and guide the agricultural ship. Had they full control of things there would be far less monotony in farm life. They would supply both the spice of variety and the sugar of sweet contentment to the boys and girls of the household.

